# A 3D-structural model of unsulfated chondroitin from high-field NMR: 4-sulfation has little effect on backbone conformation

**DOI:** 10.1016/j.carres.2009.11.013

**Published:** 2010-01-26

**Authors:** Benedict M. Sattelle, Javad Shakeri, Ian S. Roberts, Andrew Almond

**Affiliations:** a Manchester Interdisciplinary Biocentre, 131 Princess Street, Manchester, M1 7DN, UK; b Michael Smith Building, Oxford Road, Manchester, M13 9PT, UK

**Keywords:** CN, chondroitin (unsulfated), COSY, correlation spectroscopy, CS, chondriotin sulfate, CSPG, CS proteoglycan, GAG, glycosaminoglycan, GlcA, d-glucuronic acid, GalNAc, *N*-acetyl-d-galactosamine, GlcNAc, *N*-acetyl-d-glucosamine, HA, hyaluronan, HMBC, heteronuclear multiple bond coherence, HSQC, heteronuclear single quantum coherence, NMR, nuclear magnetic resonance, NOESY, nuclear Overhauser effect spectroscopy, PDB, Protein data bank, PG, proteoglycan, Chondroitin sulfate, ^15^N-isotope incorporation, Conformation, NMR, K4 polysaccharide

## Abstract

The glycosaminoglycan chondroitin sulfate is essential in human health and disease but exactly how sulfation dictates its 3D-strucutre at the atomic level is unclear. To address this, we have purified homogenous oligosaccharides of unsulfated chondroitin (with and without ^15^N-enrichment) and analysed them by high-field NMR to make a comparison published chondroitin sulfate and hyaluronan 3D-structures. The result is the first full assignment of the tetrasaccharide and an experimental 3D-model of the hexasaccharide (PDB code 2KQO). In common with hyaluronan, we confirm that the amide proton is not involved in strong, persistent inter-residue hydrogen bonds. However, in contrast to hyaluronan, a hydrogen bond is not inferred between the hexosamine OH-4 and the glucuronic acid O5 atoms across the β(1→3) glycosidic linkage. The unsulfated chondroitin bond geometry differs slightly from hyaluronan by rotation about the β(1→3) *ψ* dihedral (as previously predicted by simulation), while the β(1→4) linkage is unaffected. Furthermore, comparison shows that this glycosidic linkage geometry is similar in chondroitin-4-sulfate. We therefore hypothesise that both hexosamine OH-4 and OH-6 atoms are solvent exposed in chondroitin, explaining why it is amenable to sulfation and hyaluronan is not, and also that 4-sulfation has little effect on backbone conformation. Our conclusions exemplify the value of the 3D-model presented here and progress our understanding of glycosaminoglycan molecular properties.

## Graphical abstract

**Figure:**
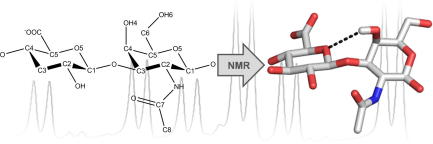

## 1 Introduction

Glycosaminoglycans (GAGs) are extracellular matrix polysaccharides that play crucial mechanical and regulatory roles in vertebrate physiology. The majority of GAGs share an anionic disaccharide repeat, comprising one uronic acid (d-glucuronic or l-iduronic) and one amino sugar (d-galactosamine or d-glucosamine). Depending on chemical features, they can be further sub-typed as hyaluronan (the only unsulfated GAG), chondroitin sulfate and heparin sulfate,^1^ which are all heterogeneous with respect to degree of polymerisation, sulfation and biological activity. In contrast to hyaluronan, the sulfated GAGs are either O- or N-linked to proteins, yielding proteoglycans, which are abundant in vertebrate digestive, endocrine, nervous, muscular, skeletal, circulatory, immune, respiratory, urinary and reproductive tissue.^2^ It is hypothesised that their diverse and enigmatic biological functions are modulated by the sulfation patterns within the constituent GAGs.^3–6^

**Figure 1:**
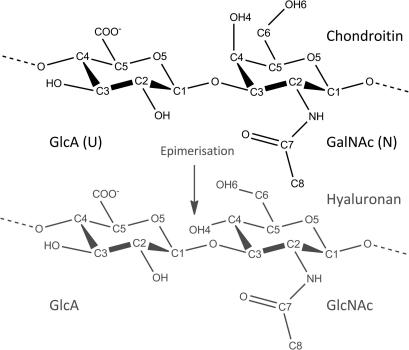
The repeating disaccharide units of unsulfated chondroitin and hyaluronan (grey). The glucuronic acid (GlcA; U) and *N*-acetyl-d-galactosamine (GalNAc; N) residues of chondroitin are joined by β(1→3) and β(1→4) glycosidic linkages. In this report the IUPAC ring numbering convention is used, that is, residues are numbered starting from the reducing end, which is capped by a hydroxyl. The adopted nomenclature for identifying carbon atoms is indicated. Hydrogen atoms assume the number of the closest bonded carbon atom.

Chondroitin sulfate (CS) comprises repeating disaccharides of d-glucuronic acid and *N*-acetyl-d-galactosamine (illustrated in Fig. 1) that can be sulfated at either the 4- or 6-hydroxyl of the galactosamine residues. The exact role of this sulfation in determining biological properties and its effect on physical properties, such as CS shape, is currently poorly understood. Furthermore, based on our current knowledge of GAG 3D-structure, and the fact that the backbones of chondroitin and hyaluronan differ by only a single stereochemical rearrangement of the 4-hydroxyl, it is difficult to explain why organisms have not evolved the ability to biosynthesise sulfated hyaluronan.

In order to improve our understanding of GAG 3D-structure and relate this to physical properties and biological function, we have previously conducted experimental and theoretical studies on defined oligosaccharides from hyaluronan and heparan sulfate. In this latest study, we turn our attention to the 3D-structure of CS, which provides the heterogeneous sulfation found in CS proteoglycans (CSPGs).^7^ These CSPGs are critical for correct neuronal development and cellular signalling,^8^ and aberrant expression of and structural modifications to CSPGs have been implicated in atherosclerosis and cancer.^7^ CS also has the potential to be the basis of new therapeutics,^9^ two CS products are in clinical use (for the treatment of burns and ulcers).^10^ Furthermore, CSPGs have attracted interest as a possible route to regenerating central nervous tissue that has been damaged by injury or disease.^11–13^

The primary barrier to structural studies of CS, and sulfated GAGs in general, has been the high level of chemical degeneracy and hence the unavailability of homogeneous material.^14^ Structural studies are further impeded by the fact that only short polymers crystallise uniformly due to their inherent flexibility^15^ and due to the characteristic overlap of NMR resonances in the spectra of oligosaccharides.^16^ One possible way to circumvent these limitations, presented here, is to initially avoid the complication of sulfate by preparing pure unsulfated chondroitin oligosaccharides for detailed NMR studies. Unsulfated chondroitin can then be used as a basis for investigating the effect of sulfation by making a comparison with the literature data or further studies.

We used capsular polysaccharide K4 from the *Escherichia coli* (O5:K4:H4) strain, which is identical (apart from a labile fructose decoration) to the unsulfated carbohydrate backbone of CS, as a preparative starting point.^17^ This strategy also allows biosynthetic incorporation of isotopes, which are useful in NMR studies (e.g., ^15^N). Here we report production and purification of natural abundance isotope and ^15^N-enriched unsulfated chondroitin oligosaccharides from K4 antigen polysaccharide, up to decasaccharides, with slight modifications to the existing protocols in order to achieve milligram quantities of homogeneous defructosylated material in minimal media. Furthermore, the similarity of pathogenic polysaccharides to GAGs is more than a coincidence,^17,18^ it is a molecular camouflage that enhances bacterial virulence. Therefore, these studies could also facilitate the development of novel anti-microbial compounds and vaccines.^19^

We previously demonstrated the value of homogeneous hyaluronan oligosaccharides,^20,21^ with and without ^15^N-isotopic enrichment, which resulted in novel sequence-specific NMR data. Again, using a similar strategy, we report a full triple-resonance (^1^H, ^13^C, ^15^N) chemical-shift assignment of the chondroitin tetrasaccharide, and provide sequence-specific insights for larger oligosaccharides. Additionally, we used ultra-high-field (900 MHz) NMR to determine a set of nuclear Overhauser effect measurements, which were used to calculate a 3D-structural model for the unsulfated chondroitin hexasaccharide in an aqueous solution.

Based on our findings, we discuss whether there is a major 3D-structural difference between the carbohydrate backbones of hyaluronan and chondroitin and consider the conformational effects of sulfation, by comparison with previous literature data. The results help clarify the role of GAG (and specifically CS) sulfation in molecular interactions and hence vertebrate physiology and provide the basis for structure-based design of novel medicines and medical devices.

## 2 Results

### 2.1 NMR and triple-resonance assignments: ^1^H, ^13^C and ^15^N

Spectra obtained with a ^1^H frequency of 600 MHz were sufficient to assign all resonances (^1^H, ^13^C and ^15^N) within the chondroitin tetramer (CN_4_) using a ^15^N-enriched sample. It was necessary to record additional data at 900 MHz to measure some scalar couplings and obtain a well-resolved NOESY spectrum on a natural abundance isotope sample for 3D-structure elucidation.

The CN_4_ all-atom assignment was achieved by taking advantage of chemical shift perturbations caused by end-effects (inter-conversion of the reducing-end hexosamine, between α- and β-anomers). The ^1^H and ^13^C atoms were fully assigned (see Table 1) using [^1^H, ^13^C]-HSQC, [^1^H, ^15^N]-HSQC, [^1^H, ^13^C]-HMBC and [^1^H, ^1^H]-COSY experiments. In Table 1 and in all subsequent tables, figures and discussion, the nomenclature for atom numbering and residue naming follows the schemes illustrated and described in Figure 1 and its footnote. Resolved ^1^H and ^13^C assignments for the residues *N*-acetyl-d-galactosamine-1 (GalNAc-1) to d-glucuronic acid-4 (GlcA-4) of CN_6_ are available as Supplementary data.

**Figure 2:**
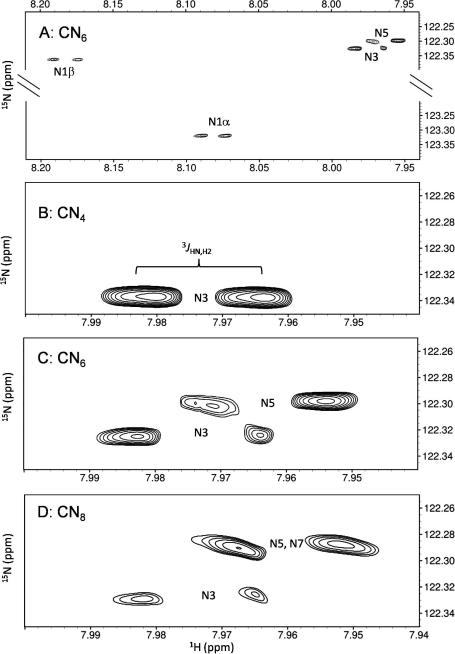
[^1^H, ^15^N]-HSQC spectra of CN oligosaccharides of different lengths. (A) Complete (broken-axis) spectrum of CN_6_. Panels (B, C and D) illustrate internal amide resonances in magnified portions of CN_4_, CN_6_ and CN_8_ spectra, respectively. The ^3^*J*_HN,H2_ coupling constant of the N3 residue in CN_4_ (also see Table 4) is annotated in panel (B). Standard errors: ^1^H ±0.001 ppm, ^15^N ±0.003 ppm. Data were acquired at 600 MHz, pH 6.0, 25 °C in 5–10% (v/v) D_2_O and referenced indirectly relative to *δ*_DSS_ (^1^H).

Enrichment with ^15^N-isotope allowed intense [^1^H, ^15^N]-HSQC spectra to be recorded rapidly, as we have shown previously in other GAGs.^20,21^ Figure 2 illustrates the examples of the [^1^H, ^15^N]-HSQC spectra for the tetra-, hexa- and octa-saccharides (CN_4_, CN_6_ and CN_8_) and the ^1^H and ^15^N chemical-shift assignments for CN_4_, CN_6_, CN_8_ and CN_10_ are detailed in Table 2. In these 2D spectra, all ^15^N nuclei were resolved at 600 MHz, apart from resonances in the N7 and N9 residues of CN_8_ and CN_10_ (they could not be significantly improved at 900 MHz). End-effects manifested as two distinct regions in the 600 MHz [^1^H, ^15^N]-HSQC NMR spectra, which are exemplified in the broken-axis CN_6_ spectrum shown in Figure 2 panel A. Both reducing-end terminal anomers were shifted downfield in the ^1^H dimension compared to the internal residues, which are magnified in panels B, C and D (for CN_4_, CN_6_ and CN_8_, respectively) in order to illustrate the resonance overlap that is a result of the very similar chemical environment of these atoms.

Due to the fact that internal ^1^H and ^13^C resonances of CN_6_ and longer oligosaccharides are indistinguishable at 600 MHz, and the fact that the ^15^N chemical shifts of the internal residues of CN_6_ and CN_8_ are almost identical (Fig. 2, panels C and D), it was considered that the chemical environment at the centre of a hexasaccharide was already similar to the polymer, as we found previously for hyaluronan oligosaccharides.^20^ This finding indicates that the hexasaccharide is the shortest length that exhibits polymeric behaviour and gives us confidence that 3D-structural observations on CN_6_ are representative of the polymer.

### 2.2 Scalar couplings and temperature coefficients

We measured scalar couplings and temperature coefficients in order to quantify ring geometry, acetimido conformation and inter-residual hydrogen bonds from the unsulfated chondroitin 3D-structure in an aqueous solution. The CN_4_^3^*J*_H,H_ values measured from 600 to 900 MHz [^1^H]–1D NMR spectra are reported in Table 3. Although we did not use these values to restrain our 3D-structural models of CN_6_, we did compare them with the values calculated from the 3D-structural models (also listed in Table 3) using the Karplus equations of Altona and Haasnoot.^22^ This comparison (vide infra) served as a retrospective quality control on the most energetically favourable 3D-structures generated.

**Figure 3:**
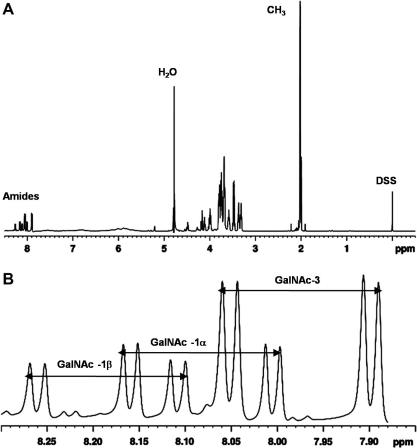
(A) The [^1^H]–1D NMR spectrum of the ^15^N-enriched unsulfated chondroitin tetrasaccharide. (B) The amide region magnified. Resonances attributed to residues GalNAc-1α, GalNAc-1β and GalNAc-3 are labelled. The large (≈90 Hz) ^1^*J*_H,N_ and small (≈10 Hz) ^3^*J*_HN,H2_ couplings are listed in Table 4. Data were acquired at 600 MHz, pH 6.0, 25 °C in 5–10% (v/v) D_2_O and referenced relative to *δ*_DSS_ (^1^H). Standard error: ^1^H ±0.001 ppm.

Figure 3 panel A illustrates a [^1^H]–1D NMR spectrum of the^15^N-enriched CN_4_ sample recorded at a ^1^H frequency of 600 MHz (no ^15^N-decoupling during acquisition). The amide region is magnified in Figure 3 panel B, showing that the amide proton resonances of the N1α, N1β and N3 residues are clearly resolved. The ^1^*J*_H,N_ and ^3^*J*_HN,H2_ coupling constants, measured from 600 MHz [^1^H]–1D spectra of the type illustrated in Figure 3 panel B, are listed in Table 4. Some of these scalar-coupling measurements were not possible for internal residues of CN_6_, CN_8_ and CN_10_, due to resonance overlap in 600 and 900 MHz [^1^H]–1D spectra. Inspection of the 2D [^1^H, ^15^N]-HSQC spectra (with ^15^N-decoupling during acquisition) for each oligosaccharide revealed similar ^3^*J*_HN,H2_ coupling constants to the tetrasaccharide but these measurements are significantly less accurate and are thus not reported here.

Table 5 lists the temperature coefficients (Δ*δ*/Δ*T*) of the exchangeable GalNAc amide proton chemical shifts in CN_4_, CN_6_, CN_8_ and CN_10_, which is a useful indicator of inter-residual hydrogen bonding, as we showed previously.^23^ The chemical shifts varied linearly with temperature in each case. With respect to all the unsulfated chondroitin oligosaccharides investigated, the Δ*δ*/Δ*T* values were in the range −6.8 to −8.4 ppb/°C.

### 2.3 A putative 3D-structure for the chondroitin sulfate backbone

**Figure 4:**
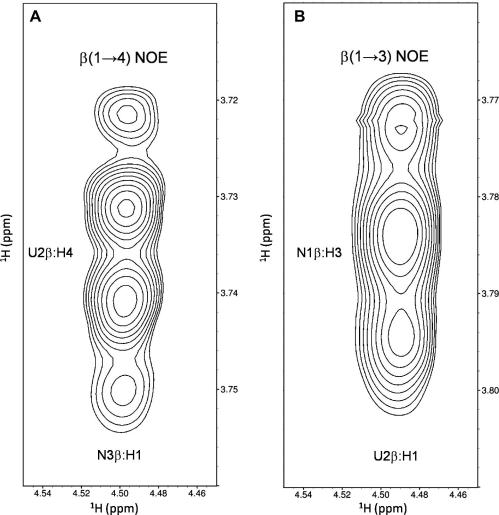
Two conformationally dependent NOEs used in the structure calculations: (A) the β(1→4) glycosidic linkage NOE, and (B) the β(1→3) glycosidic linkage NOE. Data were obtained from a 900 MHz [^1^H, ^1^H]-NOESY spectrum of natural abundance isotope CN_6_ with the axes transposed. The CN_6_ chemical shift assignments are available as Supplementary data. The sample was at pH 6.0, 25 °C in 5–10% (v/v) D_2_O and referenced relative to *δ*_DSS_ (^1^H). Standard error: ^1^H ±0.001 ppm.

A [^1^H, ^1^H]-NOESY spectrum of a natural abundance isotope CN_6_ sample was recorded at 900 MHz and used to measure nuclear Overhauser enhancements (NOEs) between protons close in spatial proximity. The ultra-high-field spectrum was used to gain extra resolution compared to 600 MHz and also to shift our NOE data further from the zero-NOE point. At 900 MHz all the NOEs were observed to be negative (i.e., had the same sign as the diagonal). We were particularly cautious only to include high-quality unambiguous NOE data in our structure calculations (see Section 4). Therefore, NOESY ^1^H–^1^H cross-peaks resulting from neighbouring protons and suffering from 3- and 4-bond COSY artefacts were not used. Table 6A lists the eight conformationally dependent (inter-residual) NOEs used in the structure calculations, binned into strong (1.8–2.7 Å) and medium (2.8–3.8 Å) distance categories. Two of the NOE cross-peaks that were used to restrain conformationally dependent distances in the structure calculations are illustrated in Figure 4. The overlapped intensities of the inter-residual β(1→4) cross-peaks (Fig. 4, panel A) were observed to be weaker than the overlapped β(1→3) cross-peaks (Fig. 4, panel B) due to the 3:2 ratio of β(1→3):β (1→4) glycosidic linkages in CN_6_ and a normalisation factor was applied. Table 6B lists the satisfactory result of back-predicting the five conformationally independent (intra-residual) reference proton–proton distances, used for calibration, from their intensities (see Section 4).

**Figure 5:**
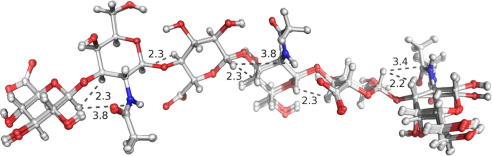
Superposition of the 25 most energetically favourable CN_6_ model conformers, PDB code 2KQO (following 750 ns restrained simulated annealing). The highlighted distances (Å) were measured from the lowest energy conformer and corresponded to the ^1^H–^1^H distance restraints.

Figure 5 illustrates the 25 lowest energy 3D-models of CN_6_ (10% of the total number generated), calculated using a restrained molecular dynamics simulated annealing protocol (described in Section 4). This ensemble has been deposited in the PDB with accession code 2KQO. It can be seen that the conformations within this most energetically favourable subset were very similar. Models ranked 2–24 in terms of total energy exhibited all atom root-mean-square-deviation values from the lowest energy conformer in the range 0.3–0.7 Å, confirming this. Distance penalties (deviations from Section 4) were negligible due to very small restraint violations for all model conformers (<0.03 Å in all cases).

Of note in Figure 5 is that all the derived 3D-models adopted an extended conformation. Furthermore, it was found that the pyranose residues remained in the ^4^*C*_1_ conformation, as expected following the application of torsional pucker-related restraints in the structure calculations (see Section 4 for details). As mentioned previously, to help assess the validity of lowest energy 3D-models, we calculated vicinal ^3^*J*_H,H_ coupling constants from H–C–C–H torsions. For this we used the Karplus equations of Altona and Haasnoot,^22^ which were modified according to the substituents. The calculated values for the first four residues of the CN_6_ 3D-model were in general agreement with the observed CN_4_ values (see Table 3). Furthermore, the observed and calculated (using the coefficients from Mobli and Almond,^24^ parenthesised) ^3^*J*_HN,H2_ scalar-coupling values of the CN_6_ lowest energy 3D-model were as follows (Hz): βN1 8.98 (10.5), βN3 9.75 (7.8) and βN5 n/d (7.8), Table 3. This result infers each of the hexosamine acetamido H2–C2–NH–HN dihedrals of the CN_6_ 3D-model to be *trans* orientated (with respect to H-2) and is consistent with the observed experimental NMR data.

Table 7 shows the average and standard deviation measurements for each glycosidic torsion in the ensemble illustrated in Figure 5. This analysis demonstrates that the reducing-end terminal β(1→3) linkage exhibited the most variability. The ensemble also showed evidence for inter-residual hydrogen bonding. For instance, with respect to the lowest energy model conformer, the GlcA O2 hydroxyl hydrogen was oriented towards, and was measured to be 2.0 Å from, the GalNAc carbonyl oxygen in two of the three β(1→3) linkages (U6:N5 and U4:N3). The β(1→4) GalNAc amide proton was hydrogen bonded to an adjacent GlcA carboxyl oxygen, with a measured distance of 2.0 Å, in both instances of this linkage.

## 3 Discussion

### 3.1 Comparison with previous NMR data on K4, chondroitin and chondroitin sulfate

Oligosaccharides of defructosylated K4 (non-^15^N-enriched) have been prepared previously using a similar method to that described here.^25,26^ Our protocols yielded oligosaccharides of a similar quality and quantity (even in minimal media) and we additionally confirmed their exact chemical composition by the absence of unassigned resonances in [^1^H, ^13^C]-HSQC NMR spectra (previous studies used HPLC and mass spectrometry). To our knowledge, NMR studies to date on K4 polysaccharide and its defructosylated derivative have been restricted to polymeric material using basic 1D NMR spectra,^27^ which were significantly less resolved than the spectra presented here and were not fully assigned. In spite of these deficiencies, our chemical-shift assignments presented in Table 1, where comparable, are in general agreement.

Due to the fact that chondroitin sulfation sites are characterised by an approximate 0.5 ppm downfield shift of H-4 and H-6 resonances for C-4 and C-6 sulfation, respectively,^28^ it was possible to further verify that we had produced unsulfated material by straightforward comparison of our chemical-shift assignments with the literature data. Recently published assignments for CS octasaccharides, acquired using ultra-high-field 900 MHz spectra, for the 6S-GalNAc-1β H6R/H6S atoms were 4.14 and 4.19 ppm.^29^ These values are indeed downfield by the expected order of magnitude (value parenthesised) of our reported observations at 3.79 (0.35) and 3.83 (0.36) ppm, respectively. The 4S-GalNAc-3 H-4 resonance, 4.18 ppm,^29^ was also downfield of our value, 3.542 ppm, by this order of magnitude (0.64 ppm).

In light of these comparisons with the literature data that are consistent with expectations and which support that unsulfated K4 material was produced and purified in this work, we propose that our assignments could be used with confidence to predict the chemical shifts for any sulfation pattern in any of the CS variants. Indeed, by providing a full triple-resonance assignment and vicinal ^3^*J*_H,H_ scalar couplings for the homogenous CN_4_ sample, many sequence-specific ^1^*J*_H,N_ and ^3^*J*_HN,H2_ scalar couplings and temperature coefficients for longer homogeneous oligosaccharides and a 3D-model of natural abundance isotope CN_6_ (vide infra), this work contributes a major advancement in our knowledge of unsulfated chondroitin properties.

### 3.2 Comparison with hyaluronan

Owing to the paradoxical structural similarity and significant functional differences of chondroitin (CN) and hyaluronan (HA), we undertook various direct comparisons of our data for CN_4_ and previous literature data for HA_4_. Resolved CN_4_ and HA_4_^1^H and ^13^C chemical shifts are compared in Table 1. Values differing by more than 0.5 ppm are highlighted. The CN_4_ hexosamine 4-position ^1^H chemical shifts were downfield (deshielded) compared to the HA_4_ values, by approximately 0.6 ppm. This observation was understood to be the manifestation of the different stereochemistries at this site. By inspection of Table 1, it was apparent that the CN_4_ hexosamine ^13^C chemical shifts were comparatively more sensitive to this structural perturbation than the ^1^H values. Significant differences in ^13^C chemical shifts (between CN_4_ and HA_4_) were evident in all hexosamine ^13^C atoms except C-1, notably the ring carbon furthest from H-4. In contrast, the C-1 atoms of both GlcA residues were shifted downfield by approximately 1 ppm in CN_4_ relative to HA_4_. This observation suggests that the stereochemical (axial/equatorial) hydroxyl rearrangement at C-4 exerts an influence on the neighbouring sugar residue, perhaps by electronic effects that are propagated through either the glycosidic linkage or hydrogen bonds.

Vicinal three-bond proton–proton (^3^*J*_H,H_) coupling constants can report on carbohydrate ring conformation. By inspection of Table 3, the effect of hexosamine 4-position epimerisation, as expected, resulted in the ^3^*J*_3,4_ and ^3^*J*_4,5_ coupling constants being approximately 5-8 Hz smaller in CN_4_ than in HA_4_. This was consistent with the small scalar coupling and *gauche* torsions (≈60°) in GalNAc residues of CN relative to the large scalar coupling and *trans* torsions (≈180°) in the *N*-acetyl-d-glucosamine (GlcNAc) residues of HA. The observed CN values were consistent with the ^4^*C*_1_ chair conformations of both GalNAc and GlcA, thereby justifying the use of ^4^*C*_1_ torsional restraints in the CN_6_ structure calculations (see Section 4). The ^1^*J*_H,N_ and ^3^*J*_HN,H2_ scalar couplings of CN (Table 4) and HA oligosaccharides were very similar. For comparison, the ^3^*J*_HN,H2_ values for CN_4_ and HA_4_^30^ (parenthesised) were 9.39 (9.47), 9.69 (9.70) and 9.75 (9.68) Hz for the hexosamine residues N1α, N1β and N3, respectively. This observation infers that both the unsulfated GAGs share a very similar geometry about this acetamido H2–C2–NH–HN torsion in an aqueous solution.

Temperature coefficients (Δ*δ*/Δ*T*) of exchangeable-proton (e.g., amide and hydroxyl) chemical shifts correlate with the presence or absence of both intra- and inter-molecular through-space interactions.^31–34^ The Δ*δ*/Δ*T* values for CN oligosaccharides are compared with previous work on HA^23^ in Table 5. Measurements larger than ±3 ppb/°C for amide protons typically denote a lack of (intra-molecular) hydrogen bonding.^35^ In common with the HA, the Δ*δ*/Δ*T* values for the CN oligosaccharides were only slightly less than those for amide protons in free exchange with water (≈−11 ppb/°C).^23^ This observation leads us to conclude that the inter-residual hydrogen bonds observed in our 3D-model of CN_6_ are not persistent but rather transient and short-lived in an aqueous solution.

### 3.3 Analysis of an NMR-based 3D-structural model of chondroitin

Due to severe overlap of resonances in carbohydrate NMR spectra there are frequently not enough NOE data to allow complete structure determination.^36^ This phenomenon is exacerbated in GAGs, which have high chemical degeneracy due to their disaccharide repeats. Therefore, despite the use of an ultra-high-field 900 MHz [^1^H, ^1^H]-NOESY spectrum, our data still suffered from a resonance overlap.

The experimental data were measured and used to generate physically allowed 3D-models of CN_6_ for comparison with the literature data on CS and HA. Due to the low number of unambiguous inter-residual [^1^H, ^1^H]-NOESY cross-peaks employed in the structure calculations, they should be considered semi-quantitative. Despite this caveat, to our knowledge this work comprises the most thorough NMR-based 3D-structural study of the unsulfated chondroitin backbone to date. We therefore considered it important to compare the inter-residual geometries of our low energy model conformers to those from HA and variously sulfated CS species for which the 3D-structure has been determined in the literature. For our analysis we used the IUPAC definition for the glycosidic phi (*φ*) and psi (*ψ*) torsions: *φ*_1–4_ = O5(N)–C1(N)–O1(N)–C4(U), *ψ*_1–4_ = C1(N)–O1(N)–C4(U)–C5(U) [GalNAc–GlcA β1→4 linkages] and *φ*_1–3_ = O5(U)–C1(U)–O1(U)–C3(N), *ψ*_1–3_ = C1(U)–O1(U)–C3(N)–C4(N) [GlcA–GalNAc β1→3 linkages].

Presently, most 3D-structures of CN and CS comprise short oligosaccharides co-crystallised with GAG-degrading lyases^37–41^ or hydrolases.^42^ To our knowledge, apart from the current work, there is just one other aqueous NMR study of homogeneous unsulfated chondriotin samples,^27^ which did not include through-space NOE data. A free polymer structure of chondroitin-4-sulfate (CS-A), determined by X-ray fibre diffraction, is available^43^ and recently several CS oligosaccharides have been studied by NMR. Chemical shifts and 2D NMR data are available for CS di-,^44^ tri-,^45^ tetra-,^44,46^ hexa-^45^ and octasaccharides.^47^ However, two combined NMR and molecular modelling studies in particular have provided the best atomistic structural insights: one of a CS pentasaccharide employing ^13^C -isotopic labelling^28^ and one study of five CS octasaccharides.^29^ We therefore chose these structures, two theoretical studies^48,49^ and the small molecule and protein-bound structures (both determined crystallographically) for comparison with our data. We also discuss our structure with reference to the solution conformation of HA_8_^50^ (PDB code 2BVK).

By inspection of Table 8, with respect to the β(1→4) linkage glycosidic torsions, our data differ at most by 7° (in both *φ* and *ψ*) from all previous studies considered with the exception of the X-ray crystallographic data (the small molecule and the enzyme-bound conformation). It has been speculated previously that crystal packing and the presence of Ca^2+^ in the small molecule structure likely explain its deviation from other literature values considered here.^28^ Indeed, compared to X-ray crystallography, the NMR method has the major advantages of allowing insight into biologically relevant (aqueous) and dynamical aspects of carbohydrate structural biology, while being free from crystal-packing artefacts. With respect to the β(1→3) linkage, and disregarding the crystallographic data, the *φ*_1–3_ values of our 3D-model are again similar to all other CN, CS and HA data considered, differing at most by 11°.

The largest differences in glycosidic linkage geometries were observed in the *ψ*_1–3_ torsions. Again, excluding the X-ray data, our values for this torsion were most similar to those of the combined NMR and molecular modelling study of a CS pentasaccharide,^28^ which employed residual-dipolar-coupling-derived orientational restraints. This comparison infers that sulfation at the hexosamine 4-position has little effect on the *ψ*_1–3_ torsion. Our *ψ*_1–3_ torsion data for the CN_6_ model differed most significantly from the solution structure of HA_8_.^50^ Both the sign and magnitude of this difference are consistent with predictions derived from a previous molecular modelling study. Almond and Sheehan^48^ hypothesised that the *ψ*_1–3_ torsion value in CN would be approximately 30° less than that in HA, due to the lack of a hydrogen bond (and incorporation of a dynamic water bridge) between the hexosamine OH-4 atom and the glucuronic acid O-5 atom across the β(1→3) linkage but that the β(1→4) linkage would remain largely unchanged. The predicted sign and magnitude of the *ψ*_1–3_ torsion difference in CN and HA is largely consistent with our data (cf. the *ψ*_1–3_ torsions of CN_6_ and HA_8_ in Table 8).

**Figure 6:**
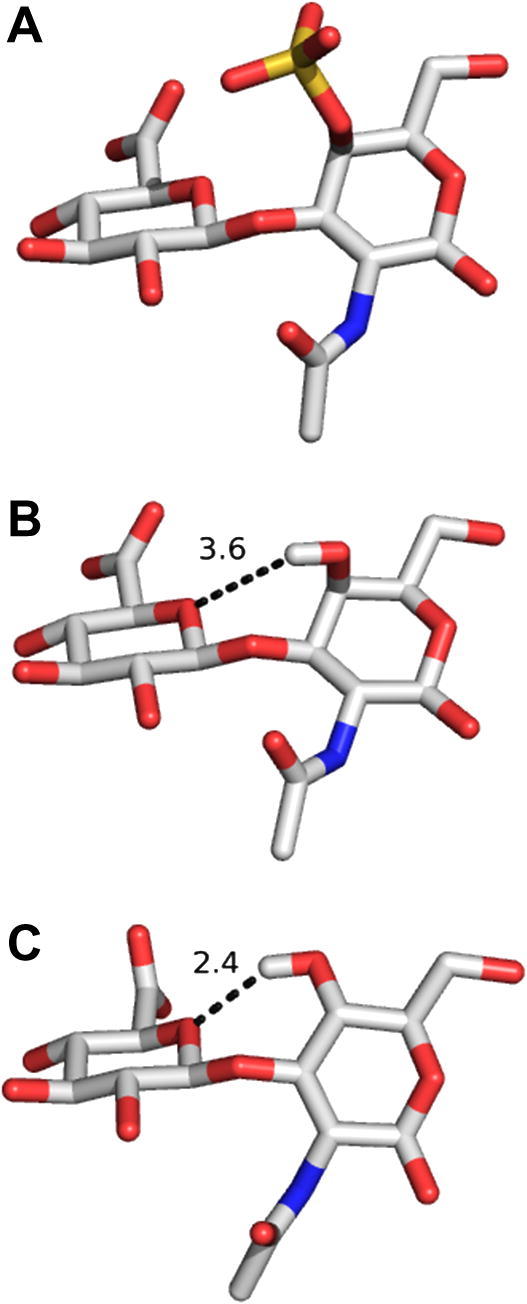
Comparison of NMR-based β(1→3) linkage geometries for: (A) chondroitin-4-sulfate, (B) unsulfated chondroitin, and (C) hyaluronan. Geometries from: (A) Yu et al.,^28^ (B) the lowest energy 3D-model of CN_6_ in this work, and (C) Almond et al.^50^ For clarity, all hydrogen atoms are hidden except for OH-4. Indicated distances (Å) are between GalNAc/GlcNAc OH-4 and GlcA O5 atoms.

Figure 6 compares β(1→3) linkage geometries from the CS pentasaccharide (panel A),^28^ the central linkage of the 3D-model of CN_6_ generated in this work (panel B) and in the aqueous 3D-structure of hyaluronan (panel C).^50^ By inspection, it is clear that the unsulfated chondroitin O4 hydroxyl hydrogen, as predicted, interacts much less strongly with the neighbouring GlcA residue O5 atom, if at all, compared to that in HA (cf. Fig. 6 panels B and C). It is therefore much more exposed to solvent than in HA.

We also note that the glycosidic linkage geometry in heparin is hypothesised to be unaffected by sulfate substituents.^51^ Although all CS structures in Table 8 were sulfated at the GalNAc 4-position, analysis of this dataset leads us to a similar conclusion, that the only significant differences between our CN_6_ 3D-models and published structures of CS (i.e., the *ψ*_1–3_ torsions in the range 11–18°) may also not be attributable to GalNAc 4-position sulfation but rather to differential solvation. Therefore, we now hypothesise that chondroitin is amenable to sulfation because the GalNAc residue of both OH-4 and OH-6 atoms are available (solvent exposed). In contrast, the hyaluronan GlcNAc 4-hydroxyl may not be suited to sulfation because it is involved in an intra-molecular hydrogen bond with the adjacent sugar residue (4-sulfation would likely disrupt the characteristic 3D-structure of hyaluronan).

In summary, the 3D-structure of the unsulfated chondroitin backbone represents a significant milestone in understanding the biological function of CS and CSPGs in the extracellular matrix. In particular, it provides an experimentally based 3D-structural platform for the investigation of all CS sulfation patterns and their interactions with proteins in physiological processes (e.g., neuronal development and cellular signalling) and disease pathologies (e.g., atherosclerosis and cancer). The 3D-model is also the basis of structural and computational strategies for developing new medicines and medical devices. In order to realise these objectives, we are working towards coarse-grained models of CS and CSPGs, which will link our microscopic 3D-structural data to macroscopic biological observations.

## 4 Experimental

### 4.1 Oligosaccharide preparation

The K4 polysaccharide was produced from cultures of the *E. coli* (05:K4:H4) strain in much the same way as described previously for the K5 antigen.^21^ The K4 serotype was confirmed by polymerase chain reaction (PCR) amplification of the *kfoC* gene encoding the K4 chondroitin polymerase gene using the primers 5′-CGGGATCCCGATGAGTATTCTTAATCTAAG3-3′ and 5′-GGAATTCCGGCCAGTCTACATGTTTATCA-3′. The nucleotide sequence of the PCR amplicon confirmed the presence of the *kfoC* gene. Bacteria were cultured to late logarithmic phase on M9-minimal media. Cells used as the source of ^15^N-enriched material were cultured on media containing 2.5 g/l ^15^N-ammonium chloride (Spectra Stable Isotopes, Columbia, MD) as the only nitrogen source. In the current work 3 g/l ferrous sulfate heptahydrate (Sigma Aldrich) was added and the glucose component was raised from 20% to 60% to increase productivity in minimal media. The K4 polysaccharide was defructosylated by acid treatment in a slightly modified version of an existing protocol.^52^ Whilst stirring at room temperature the pH was reduced to 3.0 (instead of 1.5) using 0.25 M HCl. The solution was then heated in an oil bath for 45 min at 85 °C (instead of 30 min at 80 °C). After cooling the pH was brought to 7.0 with 0.25 M sodium hydroxide and then dialysed against water for a further 24 h. The dialysed sample was lyophilised to yield defructosylated K4 polysaccharide. Digestion was achieved as in previous work.^20^ Approximately 10 mg of the defructosylated polysaccharide was dissolved in 5 ml of digest buffer (150 mM NaCl and 100 mM NaAc) and adjusted to pH 5.2 with glacial acetic acid (Fisher Scientific). Ovine Testicular hyaluronidase (100 KU, Calbiochem, Darmstadt, Germany) was added and incubated in a water bath for 3 h at 37 °C. The reaction was stopped by boiling for 10 min. Natural abundance isotope and ^15^N-enriched oligosaccharides of length 4, 6, 8 and 10 were separated (using anion-exchange chromatography) and desalted, as described previously.^21^

### 4.2 NMR sample preparation

Oligosaccharide samples for NMR spectroscopy were prepared from lyophilised material reconstituted in 500 μl MilliQ water comprising: 10% (v/v) D_2_O, 0.02% (w/v) azide (NaN_3_) 0.3 Mm and 5% DSS (4,4-dimethyl-4-silapentane-1-sulfonic acid). Approximate sample dry weights (mg) were: natural abundance isotope CN_6_, 6.6 and ^15^N-enriched CN_4_, 6.2; CN_6_, 8.2; CN_8_, 1.8; CN_10_, 7.6. Samples were adjusted to pH 6.0 to allow the observation of amide protons. The final concentration of all samples was calculated to be in the range 5–10 mM.

### 4.3 NMR spectroscopy

All spectra, with the exception of the temperature coefficient data, were recorded at 25 °C. Through-bond experiments were conducted with a ^1^H frequency of 600 MHz on a Bruker spectrometer equipped with a z-gradient TXI cryoprobe. The ^15^N-enriched tetra- and hexasaccharide [^1^H]-1D spectra were recorded with 16,384 complex points (all following numbers of points are complex), an acquisition time of 2280.7 ms and a dwell time of 69.6 μs. At 900 MHz (Varian INOVA 900), these data were recorded with 32,768 points, an acquisition time of 2949.17 ms and a dwell time of 45.5 μs. No ^15^N-decoupling was applied during acquisition.

The heteronuclear [^1^H, ^13^C]-HSQC and [^1^H, ^13^C]-HMBC spectra were standard experiments recorded on natural abundance tetra- and hexasaccharides. The [^1^H, ^15^N]-HSQC spectra were collected with acquisition times of 584.96 ms and 4096 points for ^1^H and acquisition times of 266.67 ms and 160 points for ^15^N. The dwell time and carrier frequency were set to 71.4 μs and 122.75 ppm, respectively, for these experiments. ^15^N-decoupling was applied during acquisition.

A 2D [^1^H, ^1^H]-NOESY spectrum of the unlabelled CN_6_ sample was recorded with a ^1^H frequency of 900 MHz and a mixing time of 600 ms seconds, known to be appropriate from our previous work on HA samples of the same length.^20,30^ The Varian INOVA 900 MHz NMR Spectrometer was from the Henry Wellcome Building for Biomolecular Spectroscopy, University of Birmingham, UK. A sweep width of 11,000 Hz was used in both dimensions and the respective number of points collected in the direct and indirect dimensions were 2048 and 192.

### 4.4 NMR data processing, referencing and analysis

Spectra were processed with NMRPipe^53^ and analysed with Sparky^54^ and Analysis.^55^ Proton chemical shifts were referenced relative to internal DSS. The heteronuclei were referenced indirectly. As detailed previously,^20,56,57^ appropriate linear prediction, window functions and zero-filling were used to achieve the maximum possible resolution from each dataset.

### 4.5 Structure calculations

Five conformationally independent reference NOE intensities (from cross-peaks of protons in fixed geometric arrangement, Table 6B) were used to parameterise a model relating cross-peak intensity (*I*) to proton–proton distance (*r*) using the relationship $I=\frac{k}{r^{6}}$. The constant of proportionality (*k*) was then derived from a linear plot of $\frac{1}{r^{6}}$ and intensity (*I*) for the five reference NOEs. This was employed to compute distance restraints for the eight conformationally dependent NOEs (Table 6A) which were then used to bootstrap the structure calculations described below.

The structure and energy minimised starting conformation for CN_6_ was generated using online glycam tools^58^ and then subjected to 250 rounds of restrained molecular dynamics simulated annealing comprising 3 ns each (750 ns in total) using AMBER.^59^ Non-periodic self-guided Langevin dynamics was employed; the collision frequency (velocity relaxation) term was set to 1 ps^-1^. All simulations employed a 1 fs time step and a Leapfrog integration algorithm.

Parameters of the glycam 06^60^ force-field were assigned to the hexasaccharide using the AMBER^59^ tool LEaP. As in similar previous work,^61^ force constants of 10 kcal/mol/Å^2^ and 100 kcal/mol/radians^2^ were applied to the eight NOE distance restraints and six pucker-related torsional restraints of each pyranose residue, respectively. The torsional restraints (defined on the ring heavy atoms), which precluded the formation of non-^4^*C*_1_ ring conformations during high temperature dynamics, were set alternately to 60 ± 20° and −60 ± 20°. All restraints were applied fully during heating, holding and subsequent cooling phases of molecular dynamics.

Due to the fact that explicit solvent is incompatible with the high temperatures inherent in simulated annealing, a modified generalised born continuum model^62^ was used to simulate the dielectric screening effect of water. An 18.0 Å cut-off was employed for long-range electrostatic and van der Waals interactions. The shake algorithm was applied to all bonds involving hydrogen atoms. Energetic and geometric data were recorded each ps.

The starting conformation was allowed to evolve from its initial configuration during 1 ns dynamics, throughout which it was heated linearly from 1 K to 500 K. Following thermalisation, 1 ns holding dynamics was done at 500 K, and the hexasaccharide was thereby allowed to explore regions of conformational space inaccessible at room temperature. The simulation was subsequently cooled linearly to 0 K for a further 1 ns. Final structures were geometry optimised (without restraints) using 5000 steps of steepest-descent energy minimisation followed by 5000 steps of conjugate gradient energy minimisation. Final energies were evaluated by a single point calculation using the glycam 06^60^ force-field potential.

## Figures and Tables

**Table 1:** ^1^H and ^13^C chemical-shift assignments for tetrasaccharides of unsulfated chondroitin (CN_4_) hyaluronan (HA_4_)

Ring	Reporter	HA_4_		CN_4_		ABS(CN_4_-HA_4_)	
		α	β	α	β	α	β
N1	H-1	5.155	4.713	5.205	4.665	0.050	0.048
	H-2	4.034	3.806	4.273	3.983	0.239	0.177
	H-3	3.890	3.716	3.986	3.795	0.096	0.079
	H-4	3.552	3.514	4.190	4.118	0.638	0.604
	H-5	3.869	3.472	4.104	n/d	0.235	n/d
	H6*proR*	3.792	3.750	3.727^a^	*3.652*	0.065	0.098
	H6*proS*	3.827	3.890	3.727^a^	*3.600*	0.100	0.290
	HMe	2.014	2.015	2.013	1.995	0.001	0.020

U2	H-1	4.507	4.467	4.547	4.484	0.040	0.017
	H-2	3.366	3.363	3.368		0.002	0.005
	H-3	3.585	3.580	3.582		0.003	0.002
	H-4	3.748	3.744	3.746		0.002	0.002
	H-5	3.712	3.703	3.684		0.028	0.019

N3	H-1	4.560		4.494		0.066	
	H-2	3.851		4.004		0.153	
	H-3	3.707		3.795		0.088	
	H-4	3.542		4.164		0.622	
	H-5	3.484		3.687		0.203	
	H6*proR*	3.780		3.755^a^		0.025	
	H6*proS*	3.921		3.755^a^		0.166	
	HMe	2.028		2.021		0.007	

U4	H-1	4.458		4.472		0.014	
	H-2	3.321		3.314		0.007	
	H-3	3.496		3.466		0.030	
	H-4	3.497		3.470		0.027	
	H-5	3.723		3.669		0.054	

N1	C-1	93.879	97.589	94.010	98.023	0.131	0.434
	C-2	55.817	58.454	55.121	55.142	0.696	3.312
	C-3	82.758	85.151	80.073	83.066	2.685	2.085
	C-4	71.390	71.351	71.345	70.679	0.045	0.672
	C-5	74.080	78.264	73.089	n/d	0.991	n/d
	C-6	63.398	63.567	64.103	65.672	0.705	2.105
	CMe	24.814	25.065	24.947	24.095	0.133	0.970

U2	C-1	105.675	105.818	106.812	106.907	1.137	1.089
	C-2	75.324	75.294	75.332		0.008	0.038
	C-3	76.506		76.641		0.135	
	C-4	82.781		82.502		0.279	
	C-5	79.098		79.006		0.092	

N3	C-1	103.325		103.646		0.321	
	C-2	57.049		53.794		3.255	
	C-3	85.839		83.801		2.038	
	C-4	71.357		70.494		0.863	
	C-5	78.183		77.773		0.410	
	C-6	63.390		63.925		0.535	
	CMe	25.326		25.274		0.052	

U4	C-1	105.715		106.882		1.167	
	C-2	75.578		75.580		0.002	
	C-3	78.213		78.201		0.012	
	C-4	74.560		74.644		0.084	
	C-5	78.477		78.957		0.480	

All chemical shift values are reported in units of ppm. ABS(CN_4_–HA_4_): absolute difference between CN_4_ and HA_4_ values. Highlighted (underlined) data correspond to measurements which differ in CN_4_ and HA_4_ by >0.5 ppm. Data were acquired at 600 MHz, pH 6.0, 25 °C in 5–10% (v/v) D_2_O and referenced relative to *δ*_DSS_ (^1^H). Standard errors: ^1^H ±0.001 ppm, ^13^C ±0.004 ppm. Values for HA_4_ are from Blundell et al.^30^

**Table 2:** ^1^H and ^15^N chemical-shift assignments for oligosaccharides of unsulfated chondroitin (CN) and hyaluronan (HA)

Ring	Reporter	CN_4_	HA_4_^a^	CN_6_	HA_6_^a^	CN_8_	HA_8_^a^	CN_10_	HA_10_^b^
N1α	^1^H	8.081	8.188	8.082	8.189	8.080	8.191	8.081	8.191
	^15^N	123.312	122.943	123.322	122.947	122.319	122.362	123.322	122.951

N1β	^1^H	8.184	8.271	8.183	8.271	8.183	8.181	8.180	8.271
	^15^N	122.354	122.089	122.363	122.098	122.362	122.101	122.362	122.098

N3	^1^H	7.974	8.055	7.976	8.052	7.975	8.052	7.975	8.053
	^15^N	122.337	122.134	122.326	121.996	122.329	121.996	122.333	121.993

N5	^1^H	—	—	7.963	8.042	7.961	8.042	7.960	8.040
	^15^N	—	—	122.299	122.097	122.288	121.954	122.290	121.942

N7	^1^H	—	—	—	—	n/d	8.042	n/d	8.043
	^15^N	—	—	—	—	n/d	121.907	n/d	121.950

N9	^1^H	—	—	—	—	—	—	n/d	8.042
	^15^N	—	—	—	—	—	—	n/d	122.093

All chemical-shift values are reported in units of ppm. N1–N7 denotes the GalNAc residue number according to the IUPAC classification. Data were acquired at 600 MHz, pH 6.0, 25 °C in 5–10% (v/v) D_2_O and referenced relative to *δ*_DSS_ (^1^H). Standard errors: ^1^H ±0.005 ppm, ^15^N ±0.005 ppm. n/d: value not determined for some reason, for example, due to resonance overlap.

a/b Values for HA are from Blundell et al.^20,30^

**Table 3:** ^3^*J*_H,H_ coupling constants for tetrasaccharides of unsulfated chondroitin (CN_4_) and hyaluronan (HA_4_)

Ring	^3^*J*_H,H_	CN_4_	CN_6_ CALC	HA_4_	CN_4_–HA_4_
N1α	1,2	3.72	n/c^c^	3.60	0.12
	2,3	11.20	n/c^c^	10.56	0.64
	3,4	0.62	n/c^c^	8.79	−8.17
	4,5	2.90	n/c^c^	10.06	−7.16

N1β	1,2	8.49	7.80	8.41	0.08
	2,3	10.83	8.80	n/d^a^	n/d^a^
	3,4	0.63	3.10	8.81	−8.18
	4,5	2.37	0.90	10.06	−7.69

U2α	1,2	7.94	n/c^c^	7.91	0.03
U2β	1,2	7.38	7.00	7.92	−0.54
U2α	2,3	9.54	n/c^c^	9.46	0.08
U2β	2,3	10.09	7.60	9.46	0.63
U2	3,4	7.16	7.80	8.76	−1.60
U2	4,5	9.09	8.20	9.67	−0.58

N3	1,2	8.24	6.40	8.57	−0.33
	2,3	n/d^a^	8.80	10.37	2.96
	3,4	n/d^a^	2.90	n/d^a^	n/d^a^
	4,5	4.76	0.90	10.00	−5.24

U4	1,2	8.47	7.00	7.80	0.67
	2,3	8.29	8.90	10.37	−2.08
	3,4	n/d^a^	8.00	s/c^b^	n/d^a^
	4,5	9.09	8.40	9.78	−0.69

All couplings are reported in units of hertz. Underlined values correspond to coupling constants, which differ in CN_4_ and HA_4_ by >5 Hz. Calculated (CN_6_ CALC) values were computed from the lowest energy CN_6_ conformer generated in this work using the substituent-adjusted Karplus equations of Altona and Haasnoot.^22^ Standard error on all couplings is ±0.05 Hz. CN_4_ values were measured from ^15^N-enriched 600 or 900 MHz 1D spectra. HA_4_ values are from Blundell et al.^30^

a Value not determined due to resonance overlap.

b Nuclei are strongly coupled. ^3^*J*_5,6_ and ^3^*J*_6,6_ values were not observable due to resonance overlap. CN_4_–HA_4_: the difference between CN_4_ and HA_4_ values.

c Value not calculated.

**Table 4:** ^1^*J*_H,N_ and ^3^*J*_HN,H2_ coupling constants for the unsulfated chondroitin oligosaccharides

Ring	*J*	CN_4_	CN_6_	CN_8_	CN_10_
N1α	^1^*J*_H,N_	92.74	93.13	92.74	92.71
	^3^*J*_HN,H2_	9.39	8.98	9.40	9.55

N1β	^1^*J*_H,N_	91.57	91.61	91.55	91.74
	^3^*J*_HN,H2_	9.69	9.75	9.71	9.76

N3	^1^*J*_H,N_	91.85	91.94	n/d	n/d
	^3^*J*_HN,H2_	9.75	n/d	n/d	n/d

N5	^1^*J*_H,N_	—	91.89	n/d	n/d
	^3^*J*_HN,H2_	—	n/d	n/d	n/d

N7	^1^*J*_H,N_	—	—	91.97	n/d
	^3^*J*_HN,H2_	—	—	n/d	n/d

N9	^1^*J*_H,N_	—	—	—	91.89
	^3^*J*_HN,H2_	—	—	—	n/d

All couplings are reported in units of hertz. Standard error on all couplings is ±0.05 Hz. CN_4_ values were measured from ^15^N-enriched 600 or 900 MHz 1D spectra. n/d: value not determined due to resonance overlap.

**Table 5:** Amide proton chemical shifts and temperature coefficients for unsulfated chondroitin (CN) and hyaluronan (HA) oligosaccharides of length 4, 6, 8, and 10

		5 °C	25 °C	35 °C	CN	HA^a^	CN–HA
dp	Residue	*δ*_HN_	*δ*_HN_	*δ*_HN_	Δ*δ*_HN_/Δ*T*	Δ*δ*_HN_/Δ*T*	Δ*δ*_HN_/Δ*T*
4	N1α	8.3	8.2	8.1	−8.4	−9.1	0.7
	N1β	8.4	8.3	8.2	−7.4	−7.6	0.2
	N3	8.0	7.9	7.8	−7.2	−6.9	−0.3

6	N1α	8.3	8.2	8.1	−8.0	−9.1	1.1
	N1β	8.4	8.3	8.2	−7.0	−7.6	0.6
	N3	8.2	8.1	8.0	−6.9	−6.9	0.0
	N5	8.0	7.9	7.8	−6.8	−6.7	−0.1

8	N1α	8.3	8.2	8.1	−7.9	n/d	n/d
	N1β	8.4	8.3	8.2	−7.0	n/d	n/d
	N3	n/d	n/d	n/d	n/d	n/d	n/d
	N5	n/d	n/d	n/d	n/d	n/d	n/d

10	N7	8.1	7.9	7.8	−6.9	n/d	n/d
	N1α	8.3	8.2	8.1	−8.0	−9.1	1.1
	N1β	8.4	8.3	8.2	−7.0	−7.6	0.6
	N3	n/d	n/d	n/d	n/d	n/d	n/d
	N5	n/d	n/d	n/d	n/d	n/d	n/d
	N7	n/d	n/d	n/d	n/d	n/d	n/d
	N9	8.1	7.9	7.8	−6.9	−6.9	0.0

dp: the degree of polymerisation. CN–HA: the difference between CN and HA values. *δ*_HN_ values are reported in units of ppm, Δ*δ*_HN_/Δ*T* values are reported in units of ppb/°C.

a HA data from Blundell and Almond.^23^ Standard error in temperatures: ±0.2 °C. Error in ^1^H chemical shifts ±0.001 ppm. Error in ^15^N chemical shifts ±0.003 ppm. Error in Δ*δ*_HN_/Δ*T* ≈ 0.1 ppb/°C.

**Table 6:** (A) Conformationally dependant NOEs employed in the structure calculations, (B) measured and back-predicted intra-residual reference proton–proton distances

Linkage	GalNAc	GlcA	NOE intensity
*A*			
U2:N1 (β1->3)	HN	H-1	Medium
	H-3	H-1	Strong
N3:U2 (β1->4)	H-1	H-4	Strong
U4:N3 (β1->3)	HN	H-1	Medium
	H-3	H-1	Strong
N5:U4 (β1->4)	H-1	H-4	Strong
U6:N5 (β1->3)	HN	H-1	Medium
	H-3	H-1	Strong


Residue	f2	f1	*r* (std)	*r* (pred)	*r* (pred-std)
*B*					
N-1α	H-1	H-3	3.8	3.9	0.1
N-1α	H-1	HN	3.5	3.9	0.4
N-3β	H-1	HN	2.4	2.3	0.1
N-3β	HN	H-2	3.0	3.0	0.0
U-4β	H-5	H-1	2.5	2.6	0.1

All distances (*r*) are reported in units of Å. f1 and f2: frequencies by which the [^1^H, ^1^H]-NOESY cross-peak was defined. std: distances measured from the standard energy minimised geometry of CN_6_ generated using online GLYCAM tools.^58^ pred: values predicted using the derived model relating intensity to distance discussed in Section 4. NOEs derived from non-α cross-peaks were overlapped and normalised accordingly.

**Table 7:** Average glycosidic torsions in the ensemble of 25 low energy model CN_6_ conformers

	β (1→3)	β (1→4)	β (1→3)	β (1→4)	β (1→3)
Torsion	U2–N1	N3–U2	U4–N3	N5–U4	U6–N5
*φ*	−73 (2)	−73 (0)	−73 (0)	−73 (0)	−72 (0)
*ψ*	108 (8)	−118 (1)	107 (0)	−116 (0)	109 (0)

Torsion values are reported in units of degrees. Standard deviations are given in parentheses.

**Table 8:** Comparison of glycosidic torsions in the CN_6_ model ensemble with published CS, CN and HA 3D-structures

Structure	dp	Torsion	β(1→4)	β(1→4) ABS ΔCN_6_	β(1→3)	β(1→3) ABS ΔCN_6_
NMR/simulation (CN_6_)	6	*φ*	−73	—	−72	—
		*ψ*	−117	—	108	—

NMR/simulation (CS_5_)^28^	5	*φ*	−67	6	−61	11
		*ψ*	−124	7	109	1

X-ray fibre (CS)^43^	4	*φ*	−98	25	−80	8
		*ψ*	−174	57	107	1

X-ray chondroitinase B (CS_2_)^63^	2	*φ*	−69	4	−89	17
		*ψ*	−180	63	108	0

Simulation (MM3, CS_2_)^49^	2	*φ*	−79	6	−79	7
		*ψ*	−111	6	90	18

Simulation (CHARMM, CN_4_)^48^	4	*φ*	−70	3	−70	2
		*ψ*	−120	3	90	18

NMR/simulation (CS_8_)^29^	8	*φ*	−80	7	−80	8
		*ψ*	−110	7	90	18

NMR (HA_8_)^50^	8	*φ*	−71	4	−68	8
		*ψ*	−116	7	129	20

Torsion values are reported in units of degrees. dp: the degree of polymerisation. The *φ* and *ψ* values of CN_6_ were averaged to facilitate comparison. ABS ΔCN_6_: absolute deviation from the CN_6_ value. The table was adapted from Yu et al.^28^

## Supplementary data

Supplementary data Resolved ^1^H and ^13^C assignments for residues *N*-acetyl-d-galactosamine-1 to d-glucuronic acid-4 of the ^15^N-enriched unsulfated chondroitin hexasaccharide.
