# Supplementary material for: A 3D-structural model of unsulfated chondroitin from high-field NMR: 4-sulfation has little effect on backbone conformation
Source: Carbohydr Res. 2010 Jan 26;345(2):291–302. doi: 10.1016/j.carres.2009.11.013 (PMC3098369; doi:10.1016/j.carres.2009.11.013)
Supplement: Supplementary data — Resolved 1H and 13C assignments for residues N-acetyl-d-galactosamine-1 to d-glucuronic acid-4 of the 15N-enriched unsulfated chondroitin hexasaccharide. [file mmc1.pdf]

Supporting information for:

A 3D-structural model of unsulphated chondroitin from high-field NMR: 4-sulphation has little effect on backbone conformation.

Benedict M. Sattelle[†], Javad Shakeri[†], Ian S. Roberts[‡], Andrew Almond[†,*]

[†]Manchester Interdisciplinary Biocentre, 131 Princess Street, Manchester, M1 7DN, UK

[‡]Michael Smith Building, Oxford Road, Manchester, M13 9PT, UK

*Corresponding author: Tel: 0161 306 4199 Fax: 0161 306 8918, email: Andrew.Almond@manchester.ac.uk

^{1}H chemical shift assignments for the ^{15}N-enriched unsulphated chondroitin hexasaccharide

Ring	Reporter	CN_6	
		**α**	**β**
**N1**	**H1**	5.205	4.663
	**H2**	4.272	3.982
	**H3**	3.986	3.795
	**H4**	4.189	4.112
	**H5**	4.104	n/d
	**H6*proR***	3.729	3.651
	**H6*proS***	3.729	3.598
	**HMe**	2.104	1.993
**U2**	**H1**	4.545	4.480
	**H2**	3.365	
	**H3**	3.571	
	**H4**	3.740	
	**H5**	3.682	
**N3**	**H1**	4.489	
	**H2**	4.004	
	**H3**	3.792	
	**H4**	4.161	
	**H5**	3.687	
	**H6*proR***	*3.753*	
	**H6*proS***	*3.735*	
	**HMe**	2.017	
**U4**	**H1**	4.469	
	**H2**	3.312	
	**H3**	3.469	
	**H4**	3.472	
	**H5**	3.668	

n/d: value not determined due to resonance overlap

^{13}C chemical shift assignments for the ^{15}N-enriched unsulphated chondroitin hexasaccharide

Ring	Reporter	CN_6	
		**α**	**β**
**N1**	**C1**	93.997	98.023
	**C2**	51.755	55.126
	**C3**	80.073	83.066
	**C4**	71.345	70.587
	**C5**	73.090	n/d
	**C6**	64.068	65.681
	**CMe**	24.947	24.095
**U2**	**C1**	106.812	106.951
	**C2**	75.332	
	**C3**	76.577	
	**C4**	82.455	
	**C5**	79.013	
**N4**	**C1**	103.581	
	**C2**	53.794	
	**C3**	83.115	
	**C4**	70.517	
	**C5**	77.758	
	**C6**	63.933	
	**CMe**	25.254	
**U4**	**C1**	106.960	
	**C2**	75.583	
	**C3**	78.190	
	**C4**	74.664	
	**C5**	78.987	

n/d: value not determined due to resonance overlap
